# Rate-Splitting-Based Resource Allocation in FANETs: Joint Optimization of Beam Direction, Node Pairing, Power and Time Slot

**DOI:** 10.3390/s26010224

**Published:** 2025-12-29

**Authors:** Fukang Zhao, Chuang Song, Xu Li, Ying Liu, Yanan Liang

**Affiliations:** 1 School of Electronic and Information Engineering, Beijing Jiaotong University, Beijing 100044, China; 20111049@bjtu.edu.cn (F.Z.); xli@bjtu.edu.cn (X.L.); yingliu@bjtu.edu.cn (Y.L.); 2 National Key Laboratory of Complex System Control and Intelligent Agent Cooperation, Beijing 100074, China; songchuang03@163.com

**Keywords:** flying ad hoc networks (FANETs), phased array antennas, resource allocation, constrained rate-splitting (CRS), successive convex approximation (SCA)

## Abstract

Directional flying ad hoc networks (FANETs) equipped with phased array antennas are pivotal for applications demanding high-capacity, low-latency communications. While directional beamforming extends the communication range, it necessitates the intricate joint optimization of the beam direction, power, and time-slot scheduling under hardware constraints. Existing resource allocation schemes predominantly follow two paradigms: (i) conventional physical-layer multiple access (CPMA) approaches, which enforce strict orthogonality within each beam and thus limit spatial efficiency; and (ii) advanced physical-layer techniques like rate-splitting multiple access (RSMA), which have been applied to terrestrial and omnidirectional UAV networks but not systematically integrated with the beam-based scheduling constraints of directional FANETs. Consequently, jointly optimizing the beam direction, intra-beam rate-splitting-based node pairing, transmit power, and time-slot scheduling remains largely unexplored. To bridge this gap, this paper introduces an intra-beam rate-splitting-based resource allocation (IBRSRA) framework for directional FANETs. This paper formulates an optimization problem that jointly designs the beam direction, constrained rate-splitting (CRS)-based node pairing, power control, modulation and coding scheme (MCS) selection, and time-slot scheduling, aiming to minimize the total number of time slots required for data transmission. The resulting mixed-integer nonlinear programming (MINLP) problem is solved via a computationally efficient two-stage algorithm, combining greedy scheduling with successive convex approximation (SCA) for non-convex optimization. Simulation results demonstrate that the proposed IBRSRA algorithm substantially enhances spectral efficiency and reduces latency. Specifically, for a network with 16 nodes, IBRSRA reduces the required number of transmission time slots by more than 42% compared to the best-performing baseline scheme. This confirms the significant practical benefit of integrating CRS into the resource allocation design of directional FANETs.

## 1. Introduction

Unmanned aerial vehicles (UAVs) have become a key technology across both civilian and military domains, owing to their unique advantages in mobility and deployment flexibility. In civilian applications, they are regarded as a key enabler for sixth-generation (6G) wireless networks, which are capable of providing communication coverage in complex environments such as disaster areas, dense urban regions, and remote terrains [1,2]. UAVs also play a vital role in assisting ground-based sensors for efficient data collection [3]. In the military sphere, UAVs are indispensable for reconnaissance, surveillance, and precision strike missions [4]. To efficiently execute these complex and often collaborative tasks, multiple UAVs typically form a flying ad hoc network (FANET) [5]. Such networks enable distributed coordination, enhance overall system survivability, and improve the scalability of missions.

Compared with conventional FANETs employing omnidirectional antennas, directional FANETs equipped with phased array antennas achieve a significantly extended communication range, enhanced interference suppression, and improved spectral efficiency by focusing transmitted energy into narrow beams [6,7]. However, these advantages introduce strongly coupled resource interdependencies among beam direction, transmit power, and time-slot scheduling. In practice, given the stringent hardware and cost constraints of UAV platforms, single-port phased array antennas have emerged as a widely deployed solution [8]. Specifically, directional FANETs utilizing single-port phased array antennas typically operate under a time division multiple access (TDMA)-based medium access control (MAC) protocol, adhering to a one-beam-per-slot transmission model. Such a constraint necessitates the design of optimized resource allocation schemes to enable highly efficient directional FANET communications.

### 1.1. Related Work

To address the aforementioned resource allocation challenge in directional FANETs, existing research can be broadly categorized into two complementary paradigms: (i) conventional physical-layer multiple access (CPMA)-based resource allocation, and (ii) advanced physical-layer multiple access (APMA)-based resource allocation, specifically leveraging rate-splitting multiple access (RSMA).

(i) CPMA-Based Resource Allocation: Most existing resource allocation schemes for directional FANETs adopt CPMA technologies, where only one node is permitted to transmit within a single beam, otherwise leading to severe collisions. Early studies have focused on collision-avoidance MAC designs, adopting transmission reservation and directional handshake mechanisms to reduce collision risks [9]. Subsequent studies formulate concurrent transmission scheduling as graph-theoretic problems (e.g., graph coloring) to maximize spatial reuse while satisfying interference constraints [10,11,12]. Such schemes are required to avoid inter-node/inter-beam interference by assigning orthogonal time slots or beams.

More recent research explores the joint optimization of time-slot scheduling, beam direction, and power control to further enhance network performance. For example, the authors in [13] propose a fairness-aware weighted capacity maximization framework solved via iterative dual optimization, while [14,15] introduce deep reinforcement learning (DRL) techniques to enable adaptive and dynamic resource allocation. Although these methods improve scheduling efficiency and long-term performance, they inherently assume orthogonal intra-beam transmission. Consequently, the physical-layer transmission efficiency within each beam remains fundamentally limited.

(ii) APMA-Based Resource Allocation: Non-orthogonal multiple access (NOMA) and RSMA have been widely investigated as flexible physical-layer techniques for managing multi-user interference to improve spectral efficiency in 6G networks [16,17,18]. Power-domain NOMA enables simultaneous transmission by superimposing user signals with different power levels [16]. RSMA generalizes NOMA by splitting messages into common and private parts, allowing partial interference decoding and offering improved robustness against channel uncertainty and heterogeneous link conditions [19,20].

Most existing research on RSMA has predominantly focused on terrestrial downlink and uplink communications, addressing various resource allocation challenges. In downlink scenarios, the joint optimization of user pairing, rate allocation, power control, and beamforming has been widely investigated, as seen in works such as [19,21]. The authors in [19] also design an adaptive modulation and coding scheme (MCS) for rate-splitting (RS). Corresponding studies in the uplink domain [22,23,24] have pursued a similar approach, which involves applying constrained rate-splitting (CRS) exclusively at the weaker node while performing the joint optimization of user pairing, rate allocation, and power control. Beyond conventional terrestrial systems, recent efforts have extended RSMA to high-mobility and non-terrestrial networks. For example, an orthogonal time-frequency space (OTFS)-RSMA scheme is proposed for uplink transmission, where high-mobility users are served in the delay-Doppler domain while low-mobility users operate in the time-frequency domain [25]. RSMA has also been studied in satellite communication contexts with research focusing on the joint optimization of user pairing, rate allocation, power control, and beamforming [26]. Furthermore, RSMA has been adapted for massive access and internet of things (IoT)-oriented scenarios. One research direction integrates RSMA with slotted ALOHA, introducing a preconfigured signal-to-interference-plus-noise ratio (SINR) level allocation and an adaptive traffic load mechanism to maintain throughput stability under high traffic loads [27]. Another line of work explores a UAV-assisted IoT system employing RSMA, which jointly optimizes transmission scheduling, rate allocation, transmit power, and UAV trajectory to maximize the overall system throughput [28]. Despite these advances, existing RSMA frameworks are typically developed independently of slot-based MAC protocols and do not consider the tight coupling between beam direction, intra-beam CRS-based node pairing, transmit power, MCS selection, and time-slot scheduling in directional FANETs.

While CPMA-based approaches offer structured scheduling under orthogonality constraints, and APMA-based techniques (especially RSMA) provide higher spectral efficiency in various scenarios, significant potential remains for enhancing directional FANET performance through their integrated design. To the best of our knowledge, no existing work jointly optimizes beam direction, intra-beam CRS-based node pairing, transmit power, MCS selection, and time-slot scheduling for directional FANETs. Table 1 summarizes the key distinctions between prior research and the proposed work.

### 1.2. Motivation and Contributions

Motivated by the above observations, this paper proposes an intra-beam rate-splitting-based resource allocation (IBRSRA) algorithm for phased-array-antenna-based FANETs.

The main contributions of this work are summarized as follows:

(i) This paper develops a system model that integrates directional beamforming, CRS-based intra-beam paired-node transmission, and slot-based MAC scheduling. This model explicitly captures the coupling among beam direction, intra-beam interference management, and time-slot allocation in low-latency cooperative UAV reconnaissance scenarios.
(ii) This paper formulates a mixed-integer nonlinear programming (MINLP) problem aiming to minimize the total number of time slots required to complete multi-UAV data transmission. The problem jointly considers beam direction selection, intra-beam node pairing or single-node transmission, transmit power control, and MCS selection under CRS-based transmission. To efficiently solve this problem, a two-stage optimization framework is developed, where successive convex approximation (SCA) is applied to handle non-convex intra-beam parameter optimization, which is followed by a greedy scheduling strategy to determine time-slot allocation.
(iii) Extensive simulations confirm that the proposed IBRSRA transmission mechanism substantially enhances per-slot transmission efficiency and reduces the overall delay. For a network with 16 nodes, it lowers the required number of time slots by over 42% compared to the best baseline.

### 1.3. Organization

The remainder of this paper is organized as follows. Section 2 introduces the system model. Section 3 formulates the problem of minimizing the total number of slots and presents the proposed IBRSRA algorithm. Section 4 provides the simulation parameters and performance evaluation. Finally, Section 5 concludes the paper.

## 2. System Model

### 2.1. Network Model

**Figure 1:**
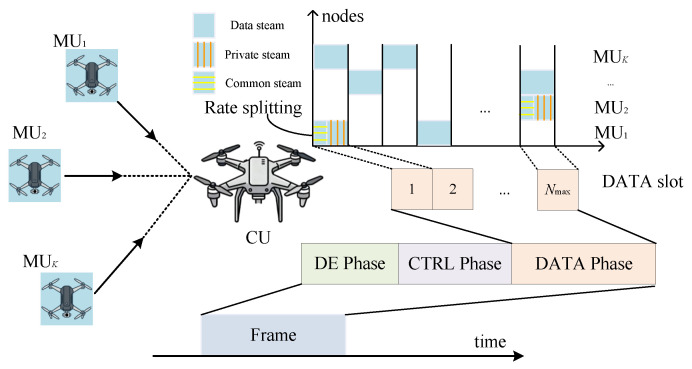
In the FANET, a central UAV and *K* mission UAVs operate cooperatively with MUs sending reconnaissance data to the CU. All UAVs are synchronized via a TDMA-based MAC protocol. Time is structured into frames, each consisting of three phases: DoA Estimation, Control, and Data, which are further divided into slots. The Control phase executes a resource allocation scheme that assigns up to $N_{\max}$ DATA slots for data transmission. Within a DATA slot, two nodes in the same beam transmit simultaneously, one of which splits its message into a common stream and a private stream.

As illustrated in Figure 1, this paper considers a cooperative UAV reconnaissance scenario, where a central UAV (CU) and *K* mission UAVs (MUs) form a FANET. Each MU k∈K≜{1,2,…,K} is required to transmit its reconnaissance data payload of $D_{k}$ bits to the CU.

All UAVs are time-synchronized and operate under a TDMA-based MAC protocol. The timeline is partitioned into frames, each consisting of three sequential phases: Direction-of-Arrival (DoA) Estimation (DE), Control (CTRL), and Data (DATA). Each phase is further divided into time slots. A resource allocation algorithm that determines the scheduling strategy and allocates up to $N_{\max}$ slots for the DATA phase is executed in the CTRL phase, where $N_{\max}$ is a predefined upper bound on the transmission horizon per frame. The operations of each phase are detailed as follows:

(i) DE Phase: The CU estimates the DoA and distances to all MUs via beacon signals, thereby acquiring the spatial prior information (including location, speed, and direction of movement) required for subsequent resource allocation. The detailed DoA estimation procedure follows our prior work [29].
(ii) CTRL Phase: Leveraging the spatial information obtained, the CU performs resource allocation for the upcoming DATA phase. This includes optimizing the parameters for intra-beam paired nodes under a CRS scheme, such as the transmit power, MCS, and receive beam direction, and determining the per-slot transmission strategy (i.e., scheduling either a paired-MU group or a single MU in each slot).
(iii) DATA Phase: According to the decisions made in the CTRL phase, the scheduled MUs transmit their reconnaissance data to the CU within the assigned time slots.

### 2.2. Antenna Model

The CU is equipped with an *M*-element uniform linear array (ULA) having an inter-element spacing of $d_{\mathrm{array}}=\lambda /2$, where λ is the signal wavelength. This phased array antenna allows the CU to form only one directive beam per time slot for either transmission or reception. The corresponding array steering vector for a signal from direction θ is given by

(1) $$a\left(\theta \right)=\frac{1}{\sqrt{M}}{\left[\begin{array}{cccc} 1, & e^{-j\pi \;\sin\pi \theta }, & \cdots , & e^{-j\pi (M-1)\;\sin\;\theta } \end{array}\right]}^{T},$$

which captures the phase progression across the array elements. The receive beamforming vector w(φ) is then designed by setting w(φ)=a(φ) to steer the main lobe toward angle φ.

For such a ULA, the far-field radiation pattern is characterized by the array factor, which is defined as the inner product between the beamforming vector and the steering vector

(2) $$AF\left(\theta ;\varphi \right)=\left|w{\left(\varphi \right)}^{H}a\left(\theta \right)\right|.$$

According to the array factor, the 3 decibel (dB) beamwidth at a steering angle φ [29] can be approximated as

(3) $$\mathrm{BW}\left(\varphi \right)\approx \frac{0.886\lambda }{Md_{\mathrm{array}}\cos\varphi }=\frac{1.772}{M\cos\varphi }.$$

The beamwidth defines the angular coverage of the main lobe. The minimum 3 dB beamwidth is achieved for broadside steering (φ=0)

(4) $${\mathrm{BW}}_{\min}=\mathrm{BW}\left(0\right)\approx \frac{1.772}{M}.$$

**Figure 2:**
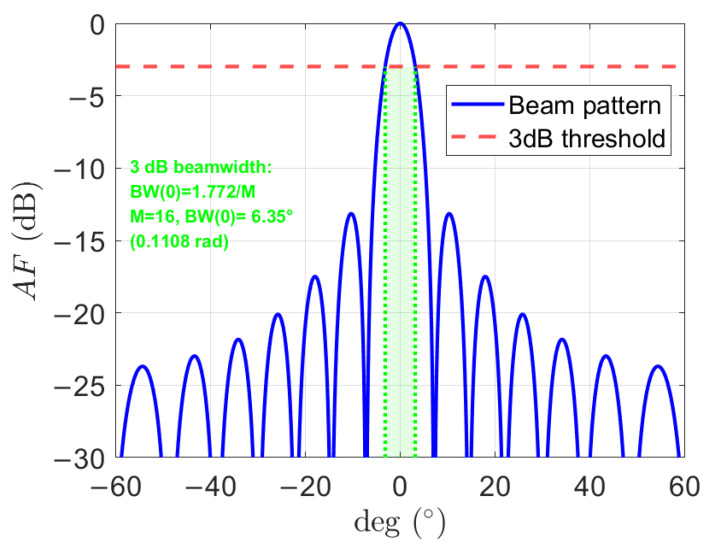
Normalized array factor of a ULA with M=16 elements.

Figure 2 shows the array factor for M=16, showing sidelobe structures and confirming that the actual 3 dB beamwidth remains close to ${\mathrm{BW}}_{\min}$ for steering angles near broadside.

Each MU employs a directional sector antenna with a constant gain of $G_{\mathrm{MU}}$ (in dB) within its main lobe and zero gain elsewhere. For analytical tractability, the beamwidth of each MU’s antenna is assumed to match the CU’s minimum 3 dB beamwidth ${\mathrm{BW}}_{\min}$, ensuring angular coverage consistency when the CU beam is steered to the broadside.

### 2.3. Mobile Model

All MUs follow a constant-velocity mobility model with fixed speed and direction within a frame [13]. In a frame comprising $N_{\max}$ DATA slots, both the speed and moving direction of each MU remain unchanged.

This paper defines a two-dimensional Cartesian coordinate system with the CU located at the origin (0,0). The *y*-axis is aligned with the normal direction of the ULA. For the *k*-th MU, its initial position at slot n=0 is expressed as

(5) $$\left(x_{k}(0),\;y_{k}(0)\right)=\left(d_{k}(0)\sin\theta_{k}(0),\;d_{k}(0)\cos\theta_{k}(0)\right),$$

where $d_{k}\left(0\right)$ is the initial distance between the MU and the CU, and $\theta_{k}\left(0\right)$ denotes the initial DoA, which is defined as the angle between the incident signal and the *y*-axis.

Let $v_{k}$ and $\vartheta_{k}$ denote the constant speed and moving direction of MU *k*, respectively, where $\vartheta_{k}$ is measured relative to the *y*-axis. The displacement per DATA slot along the *x*- and *y*-axes is given by

(6) $$\Delta x_{k}=v_{k}\sin\vartheta_{k}\;T_{\mathrm{DATA}},\;\Delta y_{k}=v_{k}\cos\vartheta_{k}\;T_{\mathrm{DATA}},$$

where $T_{\mathrm{DATA}}$ is the duration of each DATA slot.

Accordingly, the position of MU *k* in slot n≥1 updates as

(7) $$x_{k}(n)=x_{k}(0)+n\Delta x_{k},\;y_{k}(n)=y_{k}(0)+n\Delta y_{k}.$$

The corresponding Euclidean distance between MU *k* and the CU at slot *n* is given by

(8) $$d_{k}(n)=\sqrt{x_{k}^{2}\left(n\right)+y_{k}^{2}\left(n\right)},$$

and the DoA is geometrically determined by

(9) $$\theta_{k}\left(n\right)=\arcsin\;\left(\frac{x_{k}(n)}{d_{k}(n)}\right),$$

which naturally captures the angular variation induced by mobility.

### 2.4. Channel Model

This paper considers a narrowband flat-fading air-to-air (A2A) channel between the MUs and the CU, which incorporates both large-scale path loss and small-scale Rician fading [30].

#### 2.4.1. Large-Scale Path Loss

The large-scale path loss between the *k*-th MU and the CU captures the distance-dependent free-space path loss (FSPL) and random environmental fluctuations.

The distance-dependent FSPL is modeled as

(10) $$h_{\mathrm{PL}}\left(d_{k}(n)\right)={\left(\frac{\lambda }{4\pi d_{k}\left(n\right)}\right)}^{2}.$$

To account for environmental shadowing, this paper introduces a log-normal random variable $\chi_{k}(n)$ to model the shadow fading in dB. The shadow fading is given by

(11) $$h_{\mathrm{sh},k}(n)=10^{-\chi_{k}\left(n\right)/10},$$

where $\chi_{k}(n)∼N\left(0,\sigma_{\mathrm{sh}}^{2}\right)$ represents the shadowing coefficient, which is assumed to be constant within one frame and varies independently across frames.

#### 2.4.2. Small-Scale Rician Fading

To capture the dominant line-of-sight (LOS) component typical in A2A links, the small-scale fading vector is modeled as

(12) $$h_{\mathrm{SS},k}\left(n\right)=\sqrt{\frac{\beta }{\beta +1}}\;e^{j\phi_{k}\left(n\right)}a\left(\theta_{k}\left(n\right)\right)+\sqrt{\frac{1}{\beta +1}}\;h_{\mathrm{NLOS},k}\left(n\right),$$

where β is the Rician factor, $a(\theta_{k}\left(n\right))$ is the array steering vector corresponding to the DoA $\theta_{k}\left(n\right)$, and $h_{\mathrm{NLOS},k}\left(n\right)$ represents the scattered non-line-of-sight (NLOS) component.

Due to the mobility of the MUs, the channel experiences time-varying effects. The Doppler frequency shift for MU *k* is given by

(13) $$f_{d,k}=\frac{v_{k}\cos\vartheta_{k}}{\lambda },$$

which is the projection of its velocity onto the LOS direction. Consequently, the phase of the LOS component evolves as

(14) $$\phi_{k}\left(n\right)=\phi_{k}\left(n-1\right)+2\pi f_{d,k}T_{\mathrm{DATA}},\;\phi_{k}\left(0\right)∼U\left(0,2\pi \right).$$

The NLOS component is modeled as a temporally correlated Rayleigh fading process using a Jakes-inspired model

(15) $$h_{\mathrm{NLOS},k}\left(n\right)=e^{j2\pi f_{d,k}T_{\mathrm{DATA}}\cos\psi_{J,k}}\;h_{\mathrm{NLOS},k}\left(n-1\right),$$

where $\psi_{J,k}$ is a fixed realization of a random variable uniformly distributed over [0,2π), which remains constant for each MU *k*. The resulting temporal autocorrelation follows $R\left(\tau \right)=J_{0}\left(2\pi f_{d,k}\tau \right)$. The LOS and NLOS components are statistically independent.

#### 2.4.3. Composite Channel and Its Estimation

The composite A2A channel vector for MU *k* at slot *n* is therefore

(16) $$h_{k}\left(n\right)=\sqrt{h_{\mathrm{PL}}\left(d_{k}(n)\right)\;h_{\mathrm{sh},k}(n)}\;h_{\mathrm{SS},k}(n).$$

During the DE phase, the CU acquires beacon-assisted estimates of the DoA and the composite channel for each MU, which are denoted as ${\hat{\theta }}_{k}$ and ${\hat{h}}_{k}$, respectively. These estimates serve as critical inputs for the CTRL phase. The DoA estimate is subject to a bounded error modeled as [31]

(17) $${\hat{\theta }}_{k}\left(0\right)=\theta_{k}\left(0\right)+\Delta \theta_{k},\;\Delta \theta_{k}∼N\left(0,\sigma_{\theta }^{2}\right),$$

where $\sigma_{\theta }$ is the estimation error standard deviation. The corresponding initial position estimate is $\left({\hat{x}}_{k}\left(0\right),{\hat{y}}_{k}\left(0\right)\right)=\left(d_{k}\left(0\right)\sin{\hat{\theta }}_{k}\left(0\right),\;d_{k}\left(0\right)\cos{\hat{\theta }}_{k}\left(0\right)\right)$, assuming an accurate distance measurement $d_{k}\left(0\right)$ (e.g., via time of arrival). For n≥1, the estimated position is updated using the known constant velocity $v_{k}$ and direction $\vartheta_{k}$, and the DoA estimate is computed geometrically as

(18) $${\hat{\theta }}_{k}\left(n\right)=\arcsin\;\left(\frac{{\hat{x}}_{k}\left(n\right)}{{\hat{d}}_{k}\left(n\right)}\right),$$

where ${\hat{x}}_{k}(n)={\hat{x}}_{k}(0)+n\Delta x_{k}$, ${\hat{y}}_{k}(n)={\hat{y}}_{k}(0)+n\Delta y_{k}$, and ${\hat{d}}_{k}(n)=\sqrt{{\hat{x}}_{k}^{2}(n)+{\hat{y}}_{k}^{2}(n)}$. These expressions are consistent with Equations (6) and (7). This approach avoids per-slot re-estimation and is practical for TDMA-based FANETs.

The channel estimate is then modeled as

(19) $${\hat{h}}_{k}(n)=\sqrt{h_{\mathrm{PL}}\left({\hat{d}}_{k}(n)\right)\;h_{\mathrm{sh},k}(n)}\;h_{\mathrm{SS},k}\left(\beta ,{\hat{\theta }}_{k}\left(n\right)\right).$$

The effective channel for MU *k* at slot *n*, after applying the receive beamforming vector $w(\varphi_{n})$ at the CU, is given by

(20) $$h_{k}^{\mathrm{eff}}\left(n\right)=w{\left(\varphi_{n}\right)}^{H}{\hat{h}}_{k}\left(n\right)\sqrt{10^{G_{\mathrm{MU}}/10}},$$

where $G_{\mathrm{MU}}$ is the directional antenna gain of the MU.

For high-altitude FANETs operating at sub-6 GHz, ground reflections are minimal, leading to a small $\sigma_{\mathrm{sh}}$ [32]. Furthermore, with typical UAV speeds ($v_{k}\leq 28\;m/s$ [33]) and short slot durations ($T_{\mathrm{DATA}}=200\;\mu s$ [34]), both the angular drift $|{\dot{\theta }}_{k}|T_{\mathrm{DATA}}$ and the small-scale fading variation within a single slot are negligible. This results in an approximate block-fading channel that remains quasi-static within each DATA slot but evolves slowly across slots due to the cumulative effects of mobility and Doppler phase shift. Notably, for high-speed UAVs, this quasi-static approximation may break down, necessitating joint optimization with predictive positioning, which is a challenge for future work.

### 2.5. Achievable Data Rate and Remaining Data Update

For data transmission, each MU selects an MCS from a finite set $\left\{{\mathrm{MCS}}_{1},{\mathrm{MCS}}_{2},{\mathrm{MCS}}_{3},{\mathrm{MCS}}_{4}\right\}$. The spectral efficiency $Q_{q}$ (bit/s/Hz) for each MCS index *q* (q∈{1,2,3,4}) is defined as follows [35]:

- ${\mathrm{MCS}}_{1}$: QPSK with code rate 1/2, $Q_{1}=1$ bit/s/Hz;
- ${\mathrm{MCS}}_{2}$: QPSK with code rate 3/4, $Q_{2}=1.5$ bit/s/Hz;
- ${\mathrm{MCS}}_{3}$: 16-QAM with code rate 1/2, $Q_{3}=2$ bit/s/Hz;
- ${\mathrm{MCS}}_{4}$: 16-QAM with code rate 3/4, $Q_{4}=3$ bit/s/Hz.

An MCS *q* is considered feasible for a node if its linear-scale SINR satisfies $\mathrm{SINR}\geq \gamma_{q}^{\mathrm{lin}}$, where $\gamma_{q}^{\mathrm{lin}}=10^{\gamma_{q}/10}$. The dB-scale thresholds are [35] $\gamma_{1}=3.61\;\mathrm{dB}$, $\gamma_{2}=4.52\;\mathrm{dB}$, $\gamma_{3}=6.36\;\mathrm{dB}$, $\gamma_{4}=9.60\;\mathrm{dB}$.

In each slot *n*, let $S_{n}$ denote the set of scheduled MUs. The system supports two transmission modes: single-node transmission ($|S_{n}|\;=1$) and paired-node transmission ($|S_{n}|\;=2$).

#### 2.5.1. Single-Node Transmission

For a slot scheduled with a single MU *k* (i.e., $S_{n}=\left\{k\right\}$), the SINR at the CU is

(21) $${\mathrm{SINR}}_{k}^{n}=\frac{P_{k}^{n}g_{k}^{n}}{\sigma^{2}},$$

where $P_{k}^{n}$ is the transmit power of MU *k*, $\sigma^{2}$ is the additive white Gaussian noise (AWGN) power, and $g_{k}^{n}={\left|h_{k}^{\mathrm{eff}}\left(n\right)\right|}^{2}$ is the effective channel gain under the CU’s receive beam $\varphi_{n}$.

Let $q_{k}^{\mathrm{single},n}$ denote the index of the highest feasible MCS for MU *k*, satisfying ${\mathrm{SINR}}_{k}^{n}\geq \gamma_{q_{k}^{\mathrm{single},n}}^{\mathrm{lin}}$. The corresponding spectral efficiency is $Q_{q_{k}^{\mathrm{single},n}}$. The achievable data rate is then

(22) $$R_{k}^{n}=B\cdot Q_{q_{k}^{\mathrm{single},n}},$$

where *B* is the system bandwidth.

#### 2.5.2. Paired-Node Transmission

For a slot scheduled with a pair of MUs (i,j) within the same beam (i.e., $S_{n}=\left\{i,j\right\}$), the system employs CRS. To limit implementation complexity, CRS is applied only to the weaker node, which is defined as the one with the smaller effective channel gain. Without loss of generality, assume $g_{i}^{n}\leq g_{j}^{n}$, where $g_{k}^{n}={\left|h_{k}^{\mathrm{eff}}\left(n\right)\right|}^{2}$, making MU *i* the RS node. This asymmetric design is motivated by two factors: first, the RS at one node is sufficient to achieve the two-node uplink MAC capacity region [22,23,24]; second, it balances high spectral efficiency with the computational and energy constraints of UAVs [29].

Transmission Strategy: The RS node *i* splits its message into a common stream $s_{i,c}$ and a private stream $s_{i,p}$. Its total transmit power $P_{i}^{n}$ is divided as

(23) $$P_{i,c}^{n}=\alpha_{n}P_{i}^{n},\;P_{i,p}^{n}=\left(1-\alpha_{n}\right)P_{i}^{n},$$

where $\alpha_{n}\in \left(0,1\right)$ is the power-splitting factor. The stronger node *j* transmits a single stream $s_{j}$ with power $P_{j}^{n}$.

Decoding Order at CU: The CU applies successive interference cancellation (SIC) in the following fixed order: (1) decode the common stream $s_{i,c}$; (2) decode $s_{j}$ after subtracting $s_{i,c}$; (3) decode $s_{i,p}$ after subtracting both $s_{i,c}$ and $s_{j}$.

The SINR for each stream is

(24) $${\mathrm{SINR}}_{i,c}^{n}=\frac{\alpha_{n}P_{i}^{n}g_{i}^{n}}{\sigma^{2}+\left(1-\alpha_{n}\right)P_{i}^{n}g_{i}^{n}+P_{j}^{n}g_{j}^{n}},$$

(25) $${\mathrm{SINR}}_{j}^{n}=\frac{P_{j}^{n}g_{j}^{n}}{\sigma^{2}+\left(1-\alpha_{n}\right)P_{i}^{n}g_{i}^{n}},$$

(26) $${\mathrm{SINR}}_{i,p}^{n}=\frac{\left(1-\alpha_{n}\right)P_{i}^{n}g_{i}^{n}}{\sigma^{2}}.$$

MCS Selection and Achievable Rate: Each stream selects the highest feasible MCS independently based on its own SINR. Let $q_{i,c}^{n}$, $q_{j}^{n}$, and $q_{i,p}^{n}$ denote the selected MCS indices for streams $s_{i,c}$, $s_{j}$, and $s_{i,p}$, respectively. Their corresponding spectral efficiencies are $Q_{q_{i,c}^{n}}$, $Q_{q_{j}^{n}}$, and $Q_{q_{i,p}^{n}}$. The achievable data rate for MU *k* in slot *n* is

(27) $$R_{k}^{n}=\left\{\begin{array}{cc} B\left(Q_{q_{k,c}^{n}}+Q_{q_{k,p}^{n}}\right), & k=i,\\ B\;Q_{q_{k}^{n}}, & k=j. \end{array}\right.$$

Feasibility Analysis: When two nodes are paired within the same beam, their effective channel gains, denoted as $g_{i}^{n}$ and $g_{j}^{n}$, may become comparable under certain conditions, for instance, when the nodes are allocated equal initial power ($P_{i}^{n}=P_{j}^{n}$) and are located at similar distances from the UAV. In such scenarios, the fixed signal decoding order remains feasible, as its success is ultimately determined by whether the SINR at each decoding stage meets the minimum requirement of the selected MCS.

Furthermore, the algorithm incorporates a graceful degradation mechanism to sustain connectivity under challenging channel conditions:

- If the optimized intra-beam parameters yield SINRs that are insufficient to support the CRS scheme (i.e., the common stream cannot be decoded successfully), the transmission falls back to a conventional NOMA scheme while retaining the same fixed decoding order.
- If the SINRs of the common stream or the strong node’s stream fall below the lowest MCS threshold, the transmission further degrades to single-node transmission.

For the edge case where the channel gains are extremely close, an enhanced rule can be optionally applied: each node is tentatively designated as the RS node, and the corresponding joint optimization is carried out. The configuration that achieves the higher sum rate is then selected for transmission.

#### 2.5.3. Remaining Data Update

All MU nodes are assumed to have the same finite amount of data to transmit. Let $D_{k}^{\mathrm{rem}}\left(n\right)$ denote the remaining data payload of MU *k* at the beginning of slot *n* with initial condition $D_{k}^{\mathrm{rem}}\left(1\right)=D_{k}$. The payload is updated as follows

(28) $$D_{k}^{\mathrm{rem}}\left(n+1\right)=\left\{\begin{array}{cc} \max\left\{0,\;D_{k}^{\mathrm{rem}}\left(n\right)-T_{\mathrm{DATA}}R_{k}^{n}\right\}, & k\in S_{n},\\ D_{k}^{\mathrm{rem}}\left(n\right), & k\notin S_{n}, \end{array}\right.$$

where $R_{k}^{n}$ is given by (22) for single-node transmission or (27) for paired-node transmission.

## 3. Problem Formulation and Proposed Algorithm

### 3.1. Problem Formulation

Let $z_{n}\in \left\{0,1\right\}$ be a binary slot occupation indicator for $n=1,\ldots ,N_{\max}$, where $z_{n}=1$ if slot *n* is used for transmission (i.e., $|S_{n}|\;>0$), and $z_{n}=0$ otherwise. The total number of occupied slots is $\sum_{n=1}^{N_{\max}}z_{n}$.

The joint resource allocation involves optimizing over the following variables:

- Scheduling: the set $S_{n}\subseteq K$ of scheduled MUs in each slot *n* with $|S_{n}|\;\in \left\{0,1,2\right\}$.
- Transmit Powers: $P_{k}^{n}\geq 0$, the transmit power of MU *k* in slot *n* (with $P_{k}^{n}=0$ if $k\notin S_{n}$), bounded by $P_{\max}$.
- Power Splitting: for paired transmission ($|S_{n}|\;=2$), the factor $\alpha_{n}\in \left(0,1\right)$ that splits the weaker node *i*’s power into common and private parts: $P_{i,c}^{n}=\alpha_{n}P_{i}^{n}$, $P_{i,p}^{n}=\left(1-\alpha_{n}\right)P_{i}^{n}$.
- MCS Selection: the index of the selected MCS for each transmitted stream, which maps to its spectral efficiency:
  − For a single-node slot ($S_{n}=\left\{k\right\}$): $q_{k}^{\mathrm{single},n}\in \left\{1,2,3,4\right\}$, yielding spectral efficiency $Q_{q_{k}^{\mathrm{single},n}}$.
  − For a paired-node slot ($S_{n}=\left\{i,j\right\}$): $q_{i,c}^{n},q_{i,p}^{n},q_{j}^{n}\in \left\{1,2,3,4\right\}$ for the common, private, and strong node’s streams, yielding efficiencies $Q_{q_{i,c}^{n}}$, $Q_{q_{i,p}^{n}}$, and $Q_{q_{j}^{n}}$.
- Beam Direction: The CU’s receive beam angle $\varphi_{n}$ for slot *n*. For the paired-node slot ($|S_{n}|\;=2$), the receive beam angle must be within the range $\theta_{\min,ij}-\frac{{\mathrm{BW}}_{\min}}{2}\leq \varphi_{n}\leq \theta_{\max,ij}+\frac{{\mathrm{BW}}_{\min}}{2}$, where $\theta_{\min,ij}≜\min\left(\theta_{i},\theta_{j}\right)$ and $\theta_{\max,ij}≜\max\left(\theta_{i},\theta_{j}\right)$ denote the minimum and maximum DoAs of MU pair (i,j), respectively.

The goal of the optimization problem is to minimize the total transmission time, leading to the following MINLP

(29) $$\begin{array}{cc} \min_{\begin{array}{c} \left\{S_{n}\right\},\left\{P_{k}^{n}\right\},\left\{\alpha_{n}\right\},\\ \left\{q_{k}^{\cdot ,n}\right\},\left\{\varphi_{n}\right\} \end{array}}\; & \sum_{n=1}^{N_{\max}}z_{n}\;\;\;\;\;\;\;\;\;\;\;\;\;\;\;\;\;\;\; \end{array}$$

(30) $$\begin{array}{c} \;\;\;\;\;\;\;s.t.\;S_{n}\subseteq K,\;\left|S_{n}\right|\in \left\{0,1,2\right\},\;\;\;\;\;\;\;\;\;\;\;\;\;\forall n, \end{array}$$

(31) $$\begin{array}{c} \;\;\;\;\;\;\;\;\;\;0\leq P_{k}^{n}\leq P_{\max},\;\;\;\;\;\;\;\;\;\;\;\;\;\;\;\;\;\forall k\in K,\forall n, \end{array}$$

(32) $$\begin{array}{c} \;\;\;\;\;\;\;\;P_{k}^{n}=0,\;\;\;\;\;\;\;\;\;\;\;\;\;\;\;\;\;\forall k\notin S_{n},\forall n, \end{array}$$

(33) $$\begin{array}{c} \;\;\;\;\;\;\;\;\alpha_{n}\in \left(0,1\right),\;\mathrm{if}\;\left|S_{n}\right|=2,\;\;\;\;\;\;\;\;\;\;\;\;\;\;\forall n, \end{array}$$

(34) $$\begin{array}{c} \;\;\;\;\;\;\;\;\;q_{k}^{\cdot ,n}\in \left\{1,2,3,4\right\},\;\mathrm{if}\;k\in S_{n},\;\;\;\;\;\;\;\;\;\;\;\;\;\forall k,\forall n, \end{array}$$

(35) $$\begin{array}{c} \;\;\;\;\;\;\;\;\;\;{\mathrm{SINR}}_{k}^{\cdot ,n}\geq \gamma_{q_{k}^{\cdot ,n}}^{\mathrm{lin}},\;\;\;\;\;\;\;\;\;\;\;\;\;\;\;\;\;\;\forall k\in S_{n},\forall n, \end{array}$$

(36) $$\begin{array}{c} \;\;\;\;\;\;\;\;\varphi_{n}\in \left[\theta_{\min,ij}-\frac{{\mathrm{BW}}_{\min}}{2},\theta_{\max,ij}+\frac{{\mathrm{BW}}_{\min}}{2}\right],\;\mathrm{if}\;\left|S_{n}\right|=2,\forall n, \end{array}$$

(37) $$\begin{array}{c} \;\;\;\;\;\;\;\;D_{k}^{\mathrm{rem}}\left(N_{\max}+1\right)=0,\;\;\;\;\;\;\;\;\;\;\;\;\;\;\;\;\;\;\;\forall k\in K. \end{array}$$

where (30) defines the scheduling constraints. Equations (31) and (32) are the power constraints. Equation (33) defines the power splitting factor for paired transmission. Equation (34) restricts MCS selection to the predefined set. Equation (35) is the key physical-layer constraint, ensuring that the SINR of each stream supports its chosen MCS. Equation (36) defines the beam direction constraints. Equation (37) ensures that all data are transmitted by the last slot with $D_{k}^{\mathrm{rem}}\left(n\right)$ updated via (28).

This problem is a challenging MINLP due to the combinatorial scheduling variables ($S_{n}$), integer MCS selections, continuous variables ($P_{k}^{n}$, $\alpha_{n}$, $\varphi_{n}$), and the nonlinear constraint (35). Finding the global optimum is NP-hard. Therefore, in the subsequent section, this paper proposes a practical two-stage optimization algorithm to obtain efficient solutions.

### 3.2. Proposed Algorithm

To efficiently solve the challenging MINLP problem (29)–(37), this paper proposes an intra-beam rate-splitting-based resource allocation (IBRSRA) algorithm. The core idea is to decompose the original joint optimization into two interconnected subproblems: (1) intra-beam parameter optimization at slot *n* and (2) time slot scheduling.

For a given time slot *n*, the CU serves either a single MU or a paired set of MUs within a single beam. A key observation from the payload update rule (28) is that maximizing the achievable data rate transmitted in each slot directly minimizes the total number of slots required to complete all transmissions. Therefore, the per-slot objective is to maximize the achievable data rate. For a candidate single node *k*, its maximum transmittable data in a slot can be computed directly via (22) with direct beamforming. For a candidate node pair (i,j), this requires solving the intra-beam parameter optimization subproblem, which jointly determines the optimal power allocation $\left(P_{i}^{n},P_{j}^{n},\alpha_{n}\right)$, MCS indices $\left(q_{i,c}^{n},q_{i,p}^{n},q_{j}^{n}\right)$, and beam direction $\varphi_{n}$ to maximize their transmittable data sum rate.

The time slot scheduling subproblem then utilizes the results from this intra-beam optimization. By evaluating the maximum achievable data rates for all possible single nodes and node pairs, it determines the optimal schedule, i.e., which node(s) to serve in each slot, to most efficiently reduce the residual payloads $\left\{D_{k}^{\mathrm{rem}}\left(n\right)\right\}$.

**Figure 3:**
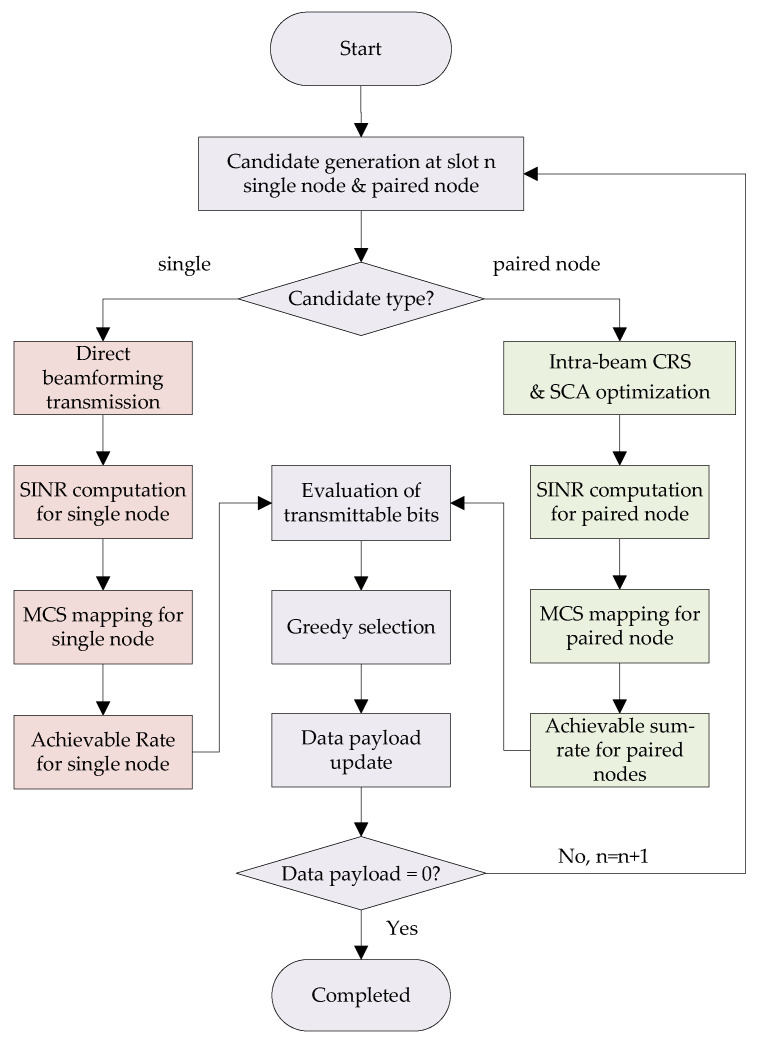
Flowchart of the proposed IBRSRA algorithm executed in each scheduled slot. The main steps involves generating transmission candidates (single nodes and pairs), solving the intra-beam parameter optimization via SCA, mapping the optimized SINR to discrete MCS, and finally selecting the candidate that maximizes the delivered data using a greedy scheduler.

Figure 3 provides a block diagram summarizing the main processing steps of the proposed IBRSRA algorithm within each scheduled slot. These steps include candidate generation, intra-beam parameter optimization, SINR-based MCS mapping (jointly performing achievable rate computation), and greedy scheduling selection.

#### 3.2.1. Intra-Beam Parameter Optimization via SCA

For a given node pair (i,j) scheduled in slot *n*, we aim to maximize their total transmission rate by jointly optimizing the transmit powers $P_{i}^{n},P_{j}^{n}$, the power-splitting factor $\alpha_{n}$, the MCS indices $\left(q_{i,c}^{n},q_{i,p}^{n},q_{j}^{n}\right)$, and the beam direction $\varphi_{n}$. The corresponding subproblem is formulated as

(38) $$\begin{array}{cc} \max_{\begin{array}{c} P_{i}^{n},P_{j}^{n},\alpha_{n},\\ q_{i,c}^{n},q_{i,p}^{n},q_{j}^{n},\varphi_{n} \end{array}} & \;R_{i}^{n}+R_{j}^{n},\;\;\;\;\; \end{array}$$

(39) $$\begin{array}{cc} s.t.\; & 0\leq P_{i}^{n},P_{j}^{n}\leq P_{\max}, \end{array}$$

(40) $$\begin{array}{cc} \; & 0<\alpha_{n}<1,\;\; \end{array}$$

(41) $$\begin{array}{c} \;\;\;\;\;\;\;\;\;\varphi_{n}\in \left[\theta_{\min,ij}-\frac{{\mathrm{BW}}_{\min}}{2},\theta_{\max,ij}+\frac{{\mathrm{BW}}_{\min}}{2}\right], \end{array}$$

where $R_{i}^{n}$ and $R_{j}^{n}$ are computed via (27) and depend on the discrete MCS selections.

To handle the combinatorial complexity introduced by the discrete MCS variables, we relax the problem by approximating the achievable rate of each stream with the continuous Shannon capacity $B\log_{2}\left(1+\mathrm{SINR}\right)$ [36,37]. The SINR expressions for the common stream, strong node’s stream, and private stream are given by (24), (25), and (26), respectively. The relaxed problem remains non-convex due to variable coupling in the SINR expressions and the logarithmic objective.

SCA linearizes non-convex terms around the current iterate, yielding a convex subproblem that can be efficiently solved per iteration [36]. This paper employs the SCA framework to solve this non-convex problem iteratively. At each iteration *l*, given the current point $x^{l}=\left(P_{i}^{n,l},P_{j}^{n,l},\alpha_{n}^{l},\varphi_{n}^{l}\right)$, a concave lower-bound surrogate for the sum rate is constructed by applying the first-order Taylor expansion to each logarithmic term. For a generic rate term $f\left(x\right)=\log_{2}\left(1+\mathrm{SINR}\left(x\right)\right)$, the expansion yields

(42) $$f\left(x\right)\geq \log_{2}\left(1+\mathrm{SINR}(x^{l})\right)+\frac{1}{\ln2}\cdot \frac{\nabla \mathrm{SINR}{\left(x^{l}\right)}^{T}\left(x-x^{l}\right)}{1+\mathrm{SINR}(x^{l})}≜\hat{f}\left(x;x^{l}\right).$$

Applying this to the three streams gives the corresponding affine approximations ${\hat{f}}_{i,c}\left(x;x^{l}\right)$, ${\hat{f}}_{j}\left(x;x^{l}\right)$, and ${\hat{f}}_{i,p}\left(x;x^{l}\right)$. The gradients $\nabla {\mathrm{SINR}}_{i,c},\nabla {\mathrm{SINR}}_{j},\nabla {\mathrm{SINR}}_{i,p}$ required for these expansions are derived in Appendix B.

Summing these approximations yields the concave surrogate objective

(43) $${\hat{R}}_{\mathrm{sum}}^{n}\left(x;x^{l}\right)=B\left[{\hat{f}}_{i,c}\left(x;x^{l}\right)+{\hat{f}}_{j}\left(x;x^{l}\right)+{\hat{f}}_{i,p}\left(x;x^{l}\right)\right].$$

Since a sum of affine functions is affine (and thus concave), ${\hat{R}}_{\mathrm{sum}}^{n}\left(x;x^{l}\right)$ is concave in x. Furthermore, the constraints (39)–(41) are all linear or box constraints, defining a convex set.

Consequently, at each SCA iteration *l*, we solve the following convex optimization problem

(44) $$\begin{array}{cc} \max_{x} & \;{\hat{R}}_{\mathrm{sum}}^{n}\left(x;x^{l}\right) \end{array}$$

(45) $$\begin{array}{cc} s.t.\; & (39),(40),(41). \end{array}$$

This convex problem can be efficiently solved using standard convex optimization solvers. The SCA algorithm iterates by solving (44)–(45) to obtain $x^{l+1}$, updating the expansion point, and repeating until convergence. The convergence criterion is based on the relative change in the surrogate objective

(46) $$\frac{\left|{\hat{R}}_{\mathrm{sum}}^{n}\left(x^{l+1};x^{l}\right)-{\hat{R}}_{\mathrm{sum}}^{n}\left(x^{l};x^{l}\right)\right|}{{\hat{R}}_{\mathrm{sum}}^{n}\left(x^{l};x^{l}\right)}\leq \epsilon ,$$

where this paper sets $\epsilon =10^{-4}$ or until a maximum number of iterations $L_{\max}$ is reached.

Let $\left(P_{i}^{n,*},P_{j}^{n,*},\alpha_{n}^{*},\varphi_{n}^{*}\right)$ denote the optimized continuous variables after SCA convergence. Then, compute the resulting SINRs ${\mathrm{SINR}}_{i,c}^{n,*},{\mathrm{SINR}}_{j}^{n,*},{\mathrm{SINR}}_{i,p}^{n,*}$ and map each to the highest feasible discrete MCS level by comparing with the thresholds $\left\{\gamma_{q}^{\mathrm{lin}}\right\}$. The selected MCS indices $q_{i,c}^{n,*},q_{i,p}^{n,*},q_{j}^{n,*}\in \left\{1,2,3,4\right\}$ for the common, private, and strong node’s streams, yield spectral efficiencies $Q_{q_{i,c}^{n,*}},Q_{q_{i,p}^{n,*}},and\;Q_{q_{j}^{n,*}}$. These efficiencies are substituted into (27) to obtain the final achievable rates $R_{i}^{n,*}$ and $R_{j}^{n,*}$.

The complete SCA procedure for a node pair is summarized in Algorithm 1. A feasible initial point $x^{0}$ (e.g., equal power splitting and a beam angle between the estimated DoAs) ensures stable convergence.
**Algorithm 1** SCA-Based Intra-Beam Optimization for Pair (i,j) in Slot *n***Require:** DoA estimates ${\hat{\theta }}_{i},{\hat{\theta }}_{j}$, distances $d_{i}^{n},d_{j}^{n}$, channel estimates ${\hat{h}}_{i}^{n},{\hat{h}}_{j}^{n}$, noise power $\sigma^{2}$, max power $P_{\max}$.**Ensure:** Optimal powers $P_{i}^{n,*},P_{j}^{n,*}$, splitting factor $\alpha_{n}^{*}$, beam angle $\varphi_{n}^{*}$, and selected MCS indices $q_{i,c}^{n,*},q_{i,p}^{n,*},q_{j}^{n,*}$.1:Initialize iteration counter l=0. Set initial point $x^{0}=\left[0.6P_{\max},\;0.6P_{\max},\;0.5,\;\left({\hat{\theta }}_{i}+{\hat{\theta }}_{j}\right)/2\right]$.2:**repeat**3:   Compute effective channel gains $g_{i}^{n}\left(\varphi^{l}\right),g_{j}^{n}\left(\varphi^{l}\right)$ using (20).4:  Compute SINRs ${\mathrm{SINR}}_{i,c}\left(x^{l}\right),{\mathrm{SINR}}_{j}\left(x^{l}\right),{\mathrm{SINR}}_{i,p}\left(x^{l}\right)$ via (24)–(26) and their gradients.5:   Construct concave surrogate ${\hat{R}}_{\mathrm{sum}}^{n}\left(x;x^{l}\right)$ via first-order Taylor expansion of all $\log_{2}\left(1+\mathrm{SINR}\right)$ terms.6:   Solve the convex problem (44) and (45) to obtain $x^{l+1}$.7:   Update l=l+1.8:**until** $\frac{|{\hat{R}}_{\mathrm{sum}}^{n}\left(x^{l};x^{l-1}\right)-{\hat{R}}_{\mathrm{sum}}^{n}\left(x^{l-1};x^{l-1}\right)|}{{\hat{R}}_{\mathrm{sum}}^{n}\left(x^{l-1};x^{l-1}\right)}\leq \epsilon$  **or** $l=L_{\max}$9:Compute final SINRs ${\mathrm{SINR}}_{i,c}^{n,*},{\mathrm{SINR}}_{j}^{n,*},{\mathrm{SINR}}_{i,p}^{n,*}$ using $x^{l}$.10:For each stream, select the highest MCS index *q* such that ${\mathrm{SINR}}^{n,*}\geq \gamma_{q}^{\mathrm{lin}}$.11:**return** $x^{l},\;q_{i,c}^{n,*},\;q_{i,p}^{n,*},\;q_{j}^{n,*}$.

Nevertheless, an empty MCS assignment occurs when the SINR obtained from the relaxed optimization falls below the minimum threshold required for the lowest-order MCS (MCS_1_). This indicates that the relaxed continuous solution becomes infeasible after discrete MCS mapping. In such cases, the transmission scheme degrades gracefully: the RSMA transmission falls back to power-domain NOMA, then to single-node transmission, or may even be skipped entirely and rescheduled in a subsequent time slot.

Furthermore, the relaxation based on the continuous Shannon capacity formula introduces an achievable rate loss (ARL) due to the discrete nature of practical MCS selection. The ARL of node *k* at slot *n* is quantified as

(47) $${\mathrm{ARL}}_{k}^{n}=\frac{R_{k,\mathrm{Shannon}}^{n}-R_{k}^{n}}{R_{k,\mathrm{Shannon}}^{n}},$$

where $R_{\mathrm{Shannon}}$ is the rate achievable under continuous SINR and $R_{\mathrm{MCS}}$ is the achieved rate from (22) and (27) under discrete SINR. The observed gap is primarily attributable to the limited granularity of the four-level MCS set adopted in this study. Employing a finer-grained MCS table, such as those specified in 5G NR [20], would effectively reduce this loss.

#### 3.2.2. Time Slot Scheduling

The scheduling subproblem determines which node(s) to serve in each time slot. It operates on the set of feasible scheduling candidates for slot *n*, which is denoted by C(n). Each candidate c∈C(n) is a subset of K that can be served by a single beam, i.e., either a single node c={k} or a node pair c={i,j}. Specifically, the following apply:

- A single node *k* is a candidate if $D_{k}^{\mathrm{rem}}\left(n\right)>0$.
- A node pair {i,j} is a candidate if both $D_{i}^{\mathrm{rem}}\left(n\right)>0$ and $D_{j}^{\mathrm{rem}}\left(n\right)>0$, and their angular separation is less than the beamwidth, ensuring they can be covered simultaneously by the same beam.

For each candidate c∈C(n), we calculate the total data it can deliver in slot *n* as

(48) $$\Delta D_{c}\left(n\right)=\left\{\begin{array}{cc} \min\;\left\{D_{k}^{\mathrm{rem}}\left(n\right),\;T_{\mathrm{DATA}}\cdot R_{k}^{n}\right\}, & \mathrm{if}\;c=\{k\},\\ \min\;\left\{D_{i}^{\mathrm{rem}}\left(n\right),\;T_{\mathrm{DATA}}\cdot R_{i}^{n}\right\}+\min\;\left\{D_{j}^{\mathrm{rem}}\left(n\right),\;T_{\mathrm{DATA}}\cdot R_{j}^{n}\right\}, & \mathrm{if}\;c=\{i,j\}, \end{array}\right.$$

where $R_{k}^{n},R_{i}^{n},$ and $R_{j}^{n}$ are the achievable rates obtained from the intra-beam optimization (Algorithm 1 for pairs, (22) for single nodes).

The scheduler adopts a greedy, per-slot maximization strategy: it selects the candidate that yields the largest immediate data delivery,

(49) $$c^{*}\left(n\right)=\arg\;\max_{c\in C(n)}\Delta D_{c}\left(n\right).$$

Ties are broken by preferring the candidate that serves more nodes (a pair over a single node). The selected candidate $c^{*}\left(n\right)$ then becomes the scheduling decision for slot *n*; i.e., we set

(50) $$S_{n}=c^{*}\left(n\right).$$

After transmission, the remaining payloads are updated via (28), and the candidate set C(n+1) for the next slot is recomputed based on the updated $\left\{D_{k}^{\mathrm{rem}}\left(n+1\right)\right\}$.

The proposed greedy scheduling rule prioritizes the candidate that maximizes the number of bits delivered per time slot. While nodes with higher SINRs tend to be scheduled more frequently in the early stages, low-SINR nodes are naturally served in subsequent stages once high-rate nodes complete their transmissions. This phenomenon can be attributed primarily to two key factors:

- All MU nodes are assumed to have identical finite data volumes of data to transmit with the overarching objective of minimizing the total number of time slots required for all nodes to complete their data transmission. Under this completion-time minimization criterion, every node must ultimately be scheduled, as failure to do so would render the system’s core objective unachievable.
- A minimum SINR guarantee is inherently embedded in the system design. Specifically, the maximum transmission distance is capped at 2 km, and a transmit power of 0.5 W ensures that the SINR resulting from single-node transmission under FSPL satisfies the minimum SINR requirement of ${\mathrm{MCS}}_{1}$ (3.61 dB). Consequently, no node is permanently precluded from transmission due to infeasible SINR conditions.

#### 3.2.3. Algorithm Summary

The complete IBRSRA algorithm, integrating the intra-beam optimization and greedy scheduling, is summarized in Algorithm 2. The algorithm proceeds slot by slot until all data payloads are cleared.
**Algorithm 2** IBRSRA Algorithm**Require:** Initial payloads ${\left\{D_{k}\right\}}_{k=1}^{K}$, DoA estimates ${\left\{{\hat{\theta }}_{k}\right\}}_{k=1}^{K}$, distances ${\left\{d_{k}\right\}}_{k=1}^{K}$, channel estimates ${\left\{{\hat{h}}_{k}\right\}}_{k=1}^{K}$.**Ensure:** Schedule ${\left\{S_{n}\right\}}_{n=1}^{N_{\mathrm{used}}}$, and corresponding parameters $\left\{P_{k}^{n},\alpha_{n},\varphi_{n},q_{k}^{\cdot ,n}\right\}$ for each scheduled slot.1:Initialize remaining data: $D_{k}^{\mathrm{rem}}\left(1\right)=D_{k}$, for all k∈K.2:Initialize slot index: n=1.3:**while **$\max_{k}D_{k}^{\mathrm{rem}}\left(n\right)>0$ **do**4:   Construct candidate set C(n):5:      Single nodes: {k} for each *k* with $D_{k}^{\mathrm{rem}}\left(n\right)>0$.6:    Node pairs: {i,j} for each i,j with $D_{i}^{\mathrm{rem}}\left(n\right)>0$, $D_{j}^{\mathrm{rem}}\left(n\right)>0$, and $|{\hat{\theta }}_{i}-{\hat{\theta }}_{j}|\;<{\mathrm{BW}}_{\min}$.7:   **for** each candidate c∈C(n) **do**8:     **if** c={k} (single node) **then**9:         Compute optimal beam direction $\varphi_{n}={\hat{\theta }}_{k}$.10:       Obtain rate $R_{k}^{n}$ via (22) with optimal power $P_{k}^{n}=P_{\max}$ and highest feasible MCS.11:        Set $\Delta D_{c}=\min\left\{D_{k}^{\mathrm{rem}}\left(n\right),\;T_{\mathrm{DATA}}\cdot R_{k}^{n}\right\}$.12:     **else if** c={i,j} (node pair) **then**13:        Run Algorithm 1 to obtain optimal $\left(P_{i}^{n},P_{j}^{n},\alpha_{n},\varphi_{n}\right)$ and rates $R_{i}^{n},R_{j}^{n}$.14:        Set $\Delta D_{c}=\min\left\{D_{i}^{\mathrm{rem}}\left(n\right),\;T_{\mathrm{DATA}}\cdot R_{i}^{n}\right\}+\min\left\{D_{j}^{\mathrm{rem}}\left(n\right),\;T_{\mathrm{DATA}}\cdot R_{j}^{n}\right\}$.15:     **end if**16:   **end for**17:   Select candidate $c^{*}=\arg\;\max_{c\in C(n)}\Delta D_{c}$. Break ties by preferring pairs over single nodes.18:   Set schedule $S_{n}=c^{*}$, and record corresponding parameters.19:   Update remaining payloads: $D_{k}^{\mathrm{rem}}\left(n+1\right)=$ result of (28) for all *k*.20:   n=n+1.21:**end while**22:Set total used slots $N_{\mathrm{used}}=n-1$.23:**return** ${\left\{S_{n}\right\}}_{n=1}^{N_{\mathrm{used}}}$, and associated parameters.

#### 3.2.4. Analysis of Computational Complexity

The computational complexity of IBRSRA primarily stems from two parts: intra-beam optimization and greedy scheduling. A detailed breakdown is provided below:

(1) Intra-Beam Parameter Optimization (per candidate pair): For each candidate node pair, the SCA algorithm solves a convex subproblem iteratively. Each subproblem has four variables ($P_{i},P_{j},\alpha ,\varphi$) and a constant number of constraints, which can be solved by an interior-point method in O(1) time per iteration. Let $L_{\mathrm{SCA}}$ denote the average number of SCA iterations needed for convergence. The complexity per pair is therefore $O(L_{\mathrm{SCA}})$. After obtaining the continuous solution, the discrete MCS mapping involves a simple lookup over four possible levels for each of the three streams, which is also O(1).

(2) Greedy Scheduling (per slot): In each slot, the algorithm evaluates all feasible candidates. Let $K_{\mathrm{rem}}\left(n\right)$ be the number of MUs with positive remaining data at slot *n*. The number of single-node candidates is $K_{\mathrm{rem}}\left(n\right)$. For node pairs, we only consider those whose angular separation is within the beamwidth, which typically reduces the number of candidate pairs from $O(K_{\mathrm{rem}}{\left(n\right)}^{2})$ to a much smaller set, which is denoted as $N_{\mathrm{pair}}\left(n\right)$. For each single node, the rate computation is closed-form (O(1)). For each candidate pair, we run Algorithm 1, incurring $O(L_{\mathrm{SCA}})$ complexity. Hence, the per-slot complexity is $O\;\left(K_{\mathrm{rem}}\left(n\right)+N_{\mathrm{pair}}\left(n\right)L_{\mathrm{SCA}}\right)$.

(3) Overall Complexity: In the worst case, where all nodes always have data and every pair is considered ($N_{\mathrm{pair}}\left(n\right)=O\left(K^{2}\right)$), and assuming the maximum possible number of slots $N_{\max}$ is used, the overall worst-case complexity becomes

(51) $$O\;\left(N_{\max}\cdot \left(K+K^{2}L_{\mathrm{SCA}}\right)\right)=O\;\left(N_{\max}K^{2}L_{\mathrm{SCA}}\right).$$

In practice, angular-based pruning significantly reduces $N_{\mathrm{pair}}\left(n\right)$, and the algorithm typically terminates in fewer than $N_{\max}$ slots. Notably, since each node has a maximum achievable transmission rate per slot, an additional pruning strategy can be incorporated: during the candidate evaluation, if either stream of a node pair reaches its maximum rate, the pair is prioritized for scheduling, further reducing the search space. Moreover, the SCA subproblems for different candidate pairs are computationally independent and can be parallelized on multi-core processors, enabling additional speedups. Thus, the proposed IBRSRA algorithm remains computationally feasible for real-time resource allocation in FANETs with a moderate number of UAVs.

To address the scalability bottleneck in large-scale node networks, a promising direction is to reformulate the resource allocation problem within the Alternating Direction Method of Multipliers (ADMM) framework. While ADMM does not change the theoretical worst-case complexity order, it enables a decomposition of the centralized pairing and resource allocation process into parallelizable local subproblems, thereby alleviating the practical computational burden and improving scalability. Key challenges for future research include designing an efficient decomposition strategy that preserves the CRS properties and establishing convergence guarantees for the resulting non-convex formulation.

#### 3.2.5. Analysis of Fairness

The fairness of the scheduling policy is evaluated using Jain’s Fairness Index [14], which is applied to the per-node completion times. While the primary objective of the IBRSRA is to minimize the total completion time, the fairness of the scheduling strategy must be carefully examined, especially since a greedy scheduler that prioritizes channel quality may, in principle, lead to the starvation of nodes with persistently poor channel conditions.

Let $T_{k}$ denote the completion time (in time slots) for node *k*, which is defined as the time slot when node *k* finishes transmitting its entire data payload. Jain’s Fairness Index *J* is computed as

(52) $$J=\frac{{\left(\sum_{k=1}^{K}T_{k}\right)}^{2}}{K\cdot \sum_{k=1}^{K}T_{k}^{2}},$$

## 4. Simulation

### 4.1. Simulation Setup

This section details the simulation setup for evaluating the proposed IBRSRA transmission mechanism (IBRSRA-TM) against three benchmark schemes: a power-domain NOMA-based mechanism (NOMA-TM) [16], a conventional TDMA-based single-node mechanism (TDMA-TM), and a deep reinforcement learning-based dynamic mechanism (DRL-TM) [15]. In NOMA-TM, paired nodes transmit without CRS with only power allocation and beamforming optimized via SCA. For the SCA procedure used in IBRSRA-TM and NOMA-TM, the convergence tolerance and maximum iteration count are set to $\epsilon =10^{-4}$ and $L_{\max}=10$, respectively [36,37]. DRL-TM employs a learning agent that adapts scheduling based on real-time channel states, using an empirically set learning rate of $10^{-4}$.

The number of MUs, *K*, varies from 8 to 32. Their angular positions $\theta_{k}$ are uniformly distributed over (−π/3,π/3), and their distances to the CU, $d_{k}$, are uniformly drawn from the range [200,2000] m, satisfying the far-field assumption [38]. The carrier frequency is 3 GHz, and each MU has a data payload of $D_{k}=2$ Kbits. The standard deviation of shadowing coefficient $\sigma_{\mathrm{sh}}$ is 1 dB [30]. The SINR thresholds for the MCS levels are $\gamma_{1}=3.61$ dB, $\gamma_{2}=4.52$ dB, $\gamma_{3}=6.36$ dB, and $\gamma_{4}=9.60$ dB [35].

Key variable parameters include the number of ULA elements M∈{8,16,24,32,40,48} (determining ${\mathrm{BW}}_{\min}$); the maximum transmit power per MU $P_{\max}\in \left\{0.5,1.0,1.5,2.0,2.5,3.0\right\}$ W; the Rician factor β (varied from 0 to 25 dB); the standard deviation of the DoA estimation error $\sigma_{\theta }\in {\left\{0,1,2,3,4,5\right\}}^{\circ }$; and the MU speed $v_{k}\in \left\{0,5,10,15,20,25,30\right\}$ m/s to model mobility. The default simulation parameters are set as follows: K=8, M=8, β=10dB, $P_{\max}=1\;W$, $\sigma_{\theta }=1^{\circ }$, $v_{k}=5\;m/s$.

Performance is assessed through 50 independent Monte Carlo simulations. The primary performance metric is the average number of time slots needed to complete all transmissions, where results for all schemes are obtained under identical channel realizations and initial conditions (i.e., the same set of random seeds). To ensure a fair and meaningful comparison, all schemes are evaluated under consistent system assumptions, including beamwidth constraints, maximum transmit power limits, MCS sets, SINR thresholds, DoA error models, channel models, and mobility configurations. No scheme is afforded additional degrees of freedom beyond its inherent transmission strategy.

### 4.2. Simulation Analysis

**Figure 4:**
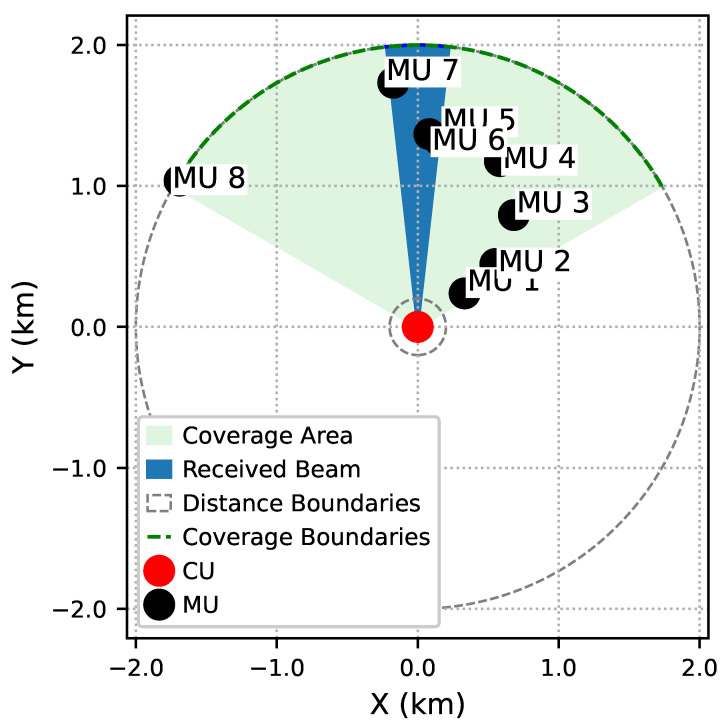
Relative positions of the CU and MUs.

**Figure 5:**
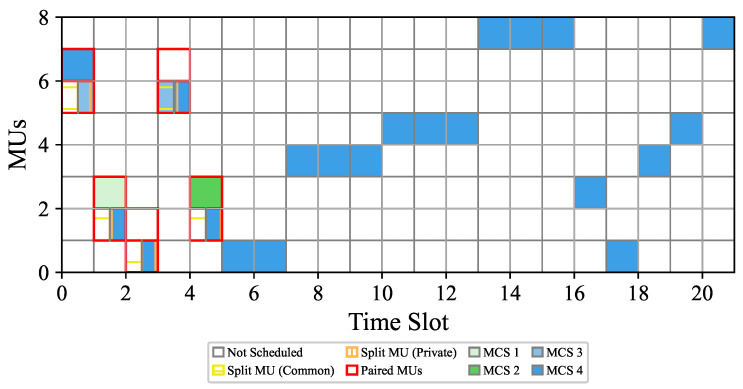
MCS and time slot scheduling results of the IBRSRA algorithm.

Figure 4 illustrates an exemplary operation of the IBRSRA-TM scheme, in which MU 5, MU 6, and MU 7 are all located within the coverage of a single beam. Under this setting, the CU executes the IBRSRA algorithm to jointly optimize intra-beam CRS and time-slot scheduling. The resulting resource allocation results are presented in Figure 5.

Figure 5 illustrates that MU 6 and MU 7 transmit simultaneously within the same beam during time slot 1. Specifically, MU 6 employs rate splitting: its common stream is unmodulated (i.e., assigned an empty MCS), while its private stream uses ${\mathrm{MCS}}_{3}$. In contrast, the stream for MU 7 is transmitted using ${\mathrm{MCS}}_{4}$. The empty common stream occurs because the corresponding SINR does not meet the minimum threshold required for the lowest available discrete MCS. Similarly, during time slots 2–5, paired MUs continue to transmit concurrently, as they are all located within the same beam (see Figure 4). From time slot 6 onward, no further simultaneous transmissions are scheduled. This is because after rate splitting, the achievable sum rate for any two nodes within the same beam no longer exceeds the rate achievable by scheduling only one of them individually in the same time slot.

**Figure 6:**
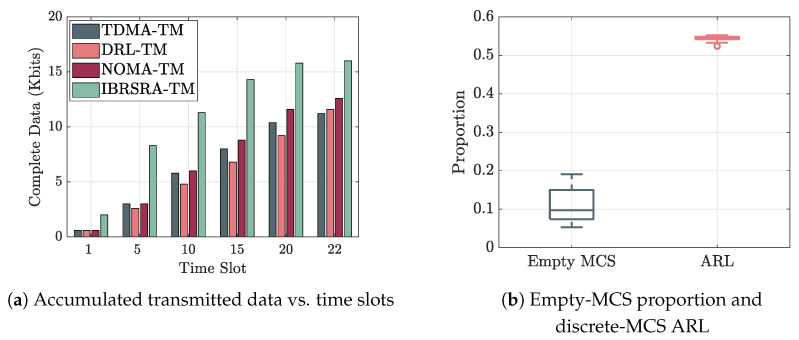
Performance comparison: (**a**) transmission completion time, and (**b**) impact of discrete MCS constraints.

Figure 6a shows that the proposed IBRSRA-TM completes data transmission for all MUs in fewer time slots than the baseline algorithms. This gain stems from CRS, which splits a single data stream into common and private parts, enabling more flexible interference management through superposition coding in the power domain and thereby achieving higher spectral efficiency.

Figure 6b further illustrates two effects of the discrete MCS constraint: the proportion of empty MCS selections and the corresponding ARL. The Shannon capacity relaxation used in the SCA leads to a mismatch where the SNR corresponding to the continuous achievable rate may fall below the minimum threshold required for the lowest MCS (${\mathrm{MCS}}_{1}$), while the actual rate under the selected discrete MCS is lower than the continuous-rate counterpart. During the execution of the IBRSRA, an empty MCS is selected in approximately 10% of cases (median over 50 runs), indicating that the algorithm can assign a suitable discrete MCS in the majority of scenarios. However, the average ARL computed for all scheduled nodes incurred by the discrete MCS set reaches about 54.5% compared to the ideal continuous rate (median over 50 runs), which is mainly due to the limited granularity of available MCS levels. Although expanding the MCS set could reduce this loss, it would also increase computational complexity. Nevertheless, even with only four MCS options, the proposed algorithm already requires fewer time slots than the baseline schemes.

**Figure 7:**
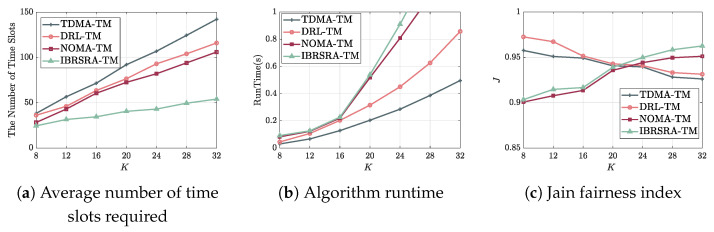
Performance versus number of MU nodes *K*: (**a**) time slots needed to complete transmission; (**b**) computational runtime; (**c**) Jain fairness index.

Figure 7a depicts the average number of time slots required to complete data transmission versus the number of MU nodes. As the number of MUs increases, all algorithms require more time slots due to the larger total data volume to be transmitted. For a given number of MUs, the algorithms can be ranked in ascending order of slot consumption as follows: IBRSRA-TM, NOMA-TM, DRL-TM, TDMA-TM. TDMA-TM employs a greedy strategy that selects the node with the highest instantaneous rate in each slot, which leads to low slot utilization. DRL-TM improves upon this by learning time-varying channel characteristics via deep reinforcement learning, achieving better long-term scheduling. NOMA-TM further increases the per-slot sum rate by enabling a power-domain superposition of multiple nodes within the same beam. The proposed IBRSRA-TM builds on NOMA-TM by incorporating rate splitting, which divides a data stream into common and private parts, thereby offering more flexible interference management and higher spectral efficiency. Quantitatively, with 16 MUs, IBRSRA-TM reduces the required time slots by 42.98% compared to NOMA-TM, by 45.67% compared to DRL-TM, and by 51.82% compared to TDMA-TM.

Figure 7b shows the algorithm runtime as a function of the number of MUs *K*. Runtime grows with the node count owing to the nonlinear increase in computational complexity. For a fixed number of nodes, the algorithms are ordered from shortest to longest runtime as TDMA-TM, DRL-TM, NOMA-TM, and IBRSRA-TM. The greedy logic of TDMA-TM incurs minimal overhead. DRL-TM introduces additional cost for the training and inference of the DRL agent. Both NOMA-TM and IBRSRA-TM rely on SCA to solve non-convex optimization problems with IBRSRA-TM requiring extra optimization variables for rate-splitting parameters (e.g., power allocation between common and private streams), which increases its computational load. Notably, when the number of MUs exceeds 16, the runtime gap between IBRSRA-TM and TDMA-TM widens sharply. Considering the trade-off between transmission efficiency and computational cost, IBRSRA-TM is most suitable for FANET reconnaissance scenarios where the number of nodes is moderate (e.g., K<16).

Figure 7c shows Jain’s Fairness Index versus the number of nodes *K*. As *K* increases, the fairness of TDMA-TM and DRL-TM decreases because more nodes compete for the same time slot, leading to a wider gap in completion times. In contrast, the fairness of NOMA-TM and IBRSRA-TM improves, since a larger node pool enhances multinode diversity and increases the probability of forming efficient node pairs, thereby balancing the transmission progress across all nodes. When K<20, the schemes are ranked in fairness as follows: DRL-TM performs best, followed by TDMA-TM, then IBRSRA-TM, and finally NOMA-TM. This is because with fewer nodes, each node in DRL-TM and TDMA-TM requires at least four time slots, resulting in relatively small differences among the nodes. In contrast, under IBRSRA-TM and NOMA-TM, certain nodes may be scheduled multiple times in a single slot due to multiplexing, which leads to a greater variation in the number of time slots needed per node and thus lower fairness. When K>20, increased node participation in slot reuse allows IBRSRA-TM to achieve the highest fairness, which is followed by NOMA-TM, then DRL-TM, and finally TDMA-TM.

**Figure 8:**
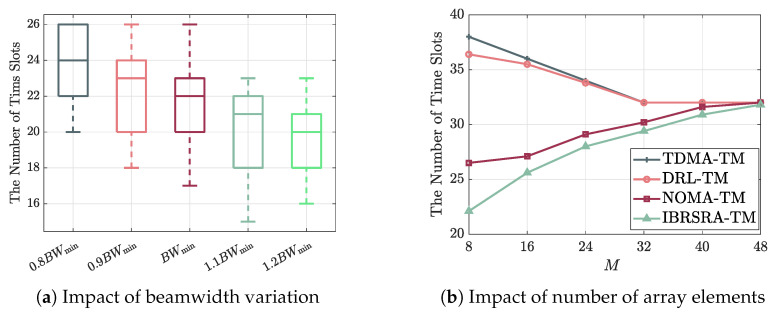
Average number of time slots required to complete data transmission under (**a**) different relative beamwidths ${\mathrm{BW}}_{\min}$ and (**b**) different numbers of array elements *M*.

Figure 8a illustrates the average number of time slots required under different beamwidths, which is expressed relative to the minimum 3 dB beamwidth BWmin. When the beamwidth is reduced to 0.8BWmin, the time slots required by IBRSRA increase by about 9.1%; conversely, widening the beam to $1.2\;{\mathrm{BW}}_{\min}$ reduces the slot count by approximately 9.1%. This occurs because a broader beam can cover more paired nodes within the same beam, thereby improving resource utilization and decreasing the total number of slots needed.

Figure 8b shows the average number of time slots versus the number of ULA array elements (*M*). As *M* increases, the 3 dB beamwidth narrows, which reduces the likelihood of covering multiple nodes within the same beam. Consequently, NOMA-TM and IBRSRA require more time slots with larger *M*. In contrast, TDMA-TM and DRL-TM exhibit a downward trend in slot count as *M* grows; this is attributed to the higher array gain provided by a narrower beam, which allows each scheduled node to achieve its maximum instantaneous rate per slot. Specifically, when M=32, the slot counts of TDMA-TM and DRL-TM reach their minimum of 32. As *M* increases further (e.g., M=48), the beamwidth of NOMA-TM and IBRSRA becomes so narrow that each beam effectively covers only a single node, causing their required slot counts to converge to those of TDMA-TM and DRL-TM.

**Figure 9:**
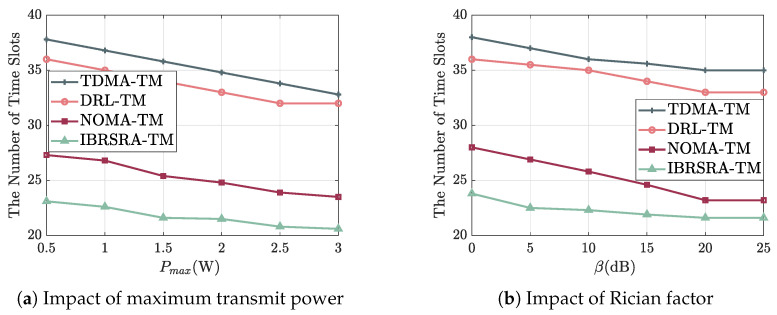
Average number of time slots required to complete data transmission under (**a**) varying maximum transmit power $P_{\max}$ and (**b**) varying Rician factor β.

Figure 9a plots the number of required time slots versus the maximum transmit power $P_{\max}$. As $P_{\max}$ increases, the slot count for all algorithms decreases monotonically, because higher transmit power improves the SINR and enables the selection of higher-order MCSs, thereby raising the per-slot throughput. For any given power level, IBRSRA-TM achieves the lowest slot count, which is followed by NOMA-TM, DRL-TM, and TDMA-TM. This performance gap originates from their transmission principles: TDMA-TM schedules only one node per slot, whereas NOMA-TM allows two nodes to transmit simultaneously via power-domain superposition. IBRSRA-TM further incorporates CRS, which provides more flexible interference management and MCS adaptation than pure NOMA, leading to higher spectral efficiency. When the transmit power reaches 3 W, both TDMA-TM and DRL-TM close their minimum possible slot counts under the given system setup.

Figure 9b shows the number of time slots required under different Rician factors (β). A larger β reduces the slot count for all schemes, as the channel becomes increasingly dominated by the strong LOS component. For a fixed β, TDMA-TM requires the most slots, while IBRSRA-TM consistently uses the fewest. This result confirms that IBRSRA-TM can effectively exploit the available channel state to jointly optimize power allocation, beam direction, and MCS selection. When β exceeds 20 dB, the channel is essentially deterministic, and the slot counts of all algorithms converge to stable minimum values.

**Figure 10:**
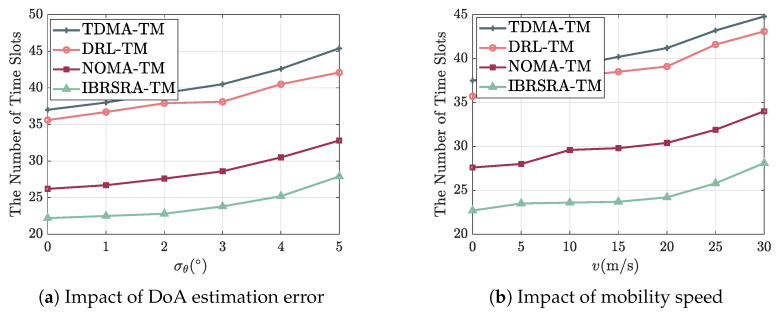
Average number of time slots required under (**a**) different DoA estimation errors $\sigma_{\theta }$ and (**b**) different MU mobility speeds *v*.

Figure 10a shows the number of time slots required under different levels of DoA estimation error. As the error increases, the slot count rises for all algorithms because misalignment reduces the beamforming gain and degrades the SINR. Across the range of errors, IBRSRA-TM consistently requires the fewest slots. This is due to its embedded CRS mechanism, which offers more flexible interference management and thus sustains higher per-slot spectral efficiency even under imperfect beam steering. When the angular deviation stays below $3^{{}^{\circ}}$, the increase in slot count remains mild, indicating that all algorithms are reasonably robust to small DoA estimation inaccuracies.

Figure 10b presents the slot count versus the mobility speed of MUs. Higher speeds lead to larger required slot numbers for all schemes, mainly because of increased beam misalignment (due to faster channel variations) and larger Doppler spread, both of which reduce the effective SINR. Again, IBRSRA-TM maintains the lowest slot count owing to its ability to adapt the CRS strategy to the changing channel. At speeds below 20 m/s, the performance degradation is gradual, demonstrating that the algorithms remain effective in low mobility regimes.

## 5. Conclusions

This paper proposes an IBRSRA scheme for single-port phased array antennas in FANETs. To improve the single-beam transmission rate, a CRS technique is incorporated, which systematically models and analyzes the minimum transmission time required for predefined data volumes. To minimize this transmission time, an MINLP problem is formulated to jointly optimize beam direction, CRS-based node pairing, transmit power, and time-slot allocation. This problem is solved via a two-stage optimization framework integrating greedy search and SCA. Extensive simulation results validate that the proposed IBRSRA outperforms state-of-the-art baseline algorithms in terms of lower time-slot consumption.

However, this paper has several limitations that merit attention. First, the proposed algorithm operates in dedicated control time slots with optimization results transmitted to MUs via control channels; however, the overhead associated with these control slots is not analyzed in detail. Second, out-of-network interference is not taken into account. Third, the channel state is assumed to be slowly time-varying, whereas UAVs with higher mobility may necessitate low-complexity dynamic resource allocation strategies to adapt to rapid channel fluctuations. Future work will focus on addressing these limitations and further enhancing the communication efficiency of FANETs in UAV reconnaissance missions.

## Figures and Tables

**Table 1:** Comparison of optimization methods and application scenarios between existing works and this work.

Literature	Optimization Dimensions					Application Scenario
	Beam Direction	Rate Splitting	Power Control	MCS Selection	Time-Slot Scheduling	
[9,10,11,12]	✓	×	×	×	✓	Directional FANET
[13,14,15]	✓	×	✓	×	✓	Directional FANET
[19]	×	✓	✓	✓	×	6G Downlink
[21,26]	✓	✓	✓	×	×	Terrestrial 6G/Satellite
[22,23,24,25]	×	✓	✓	×	×	6G Uplink
[27,28]	×	✓	✓	×	✓	Omnidirectional UAV-IoT
This Work	✓	✓	✓	✓	✓	Directional FANET

Notes: ✓ indicates supported optimization dimension; × indicates not considered.

## Data Availability

Data are contained within the article.

## Abbreviations

The following abbreviations are used in this manuscript:

FANET	Flying ad hoc network
RS	Rate-splitting
CRS	Constrained rate-splitting
IBRSRA	Intra-beam rate-splitting-based resource allocation
SCA	Successive convex approximation
UAV	Unmanned aerial vehicle
6G	Sixth-generation
RSMA	Rate-splitting multiple access
MCS	Modulation and coding scheme
MINLP	Mixed-integer non-linear programming
CU	Central UAV
MU	Mission UAV
TDMA	Time-division multiple access
MAC	Media access control
DE	DOA estimation
CTRL	Control
SIC	Successive interference cancellation
ULA	Uniform linear array
CSI	Channel state information
LOS	Line-of-sight
SINR	Signal-to-interference-plus-noise ratio
SNR	Signal-to-noise ratio
NOMA	Non-orthogonal multiple access

## Appendix A. Gradient of Channel Gain

This appendix derives the gradient of the effective channel gain with respect to the beam steering angle φ, which is a key component for the SCA algorithm in Section 3. Consistent with the system model, all channels use the estimated ${\hat{h}}_{k}$.

For a ULA with *M* elements, the normalized steering vector is a(φ). Its derivative is

$$\frac{\partial a(\varphi )}{\partial \varphi }=-j\pi \cos\varphi \cdot Aa\left(\varphi \right),$$

where A=diag(0,1,…,M−1).

The effective scalar channel is $h_{k}^{\mathrm{eff}}\left(\varphi \right)=\sqrt{G}\cdot a{\left(\varphi \right)}^{H}{\hat{h}}_{k}$, with $G=10^{G_{\mathrm{MU}}/10}$. Its gradient is

$$\frac{\partial h_{k}^{\mathrm{eff}}\left(\varphi \right)}{\partial \varphi }=\sqrt{G}\cdot j\pi \cos\varphi \cdot a{\left(\varphi \right)}^{H}A{\hat{h}}_{k}.$$

The channel gain $g_{k}\left(\varphi \right)={\left|h_{k}^{\mathrm{eff}}\left(\varphi \right)\right|}^{2}$ has the following gradient:

∂gk(φ)∂φ=2πcosφ·G·Ima(φ)HAh^kh^kHa(φ),

where Im[·] extracts the imaginary part.

## Appendix B. Gradients of the SINR Expressions

This appendix provides the gradients of the three SINRs with respect to the optimization variables $x=(P_{i},P_{j},\alpha ,\varphi )$ for the SCA algorithm. For brevity, the slot index *n* is omitted, and all variables are per-slot scalars. The effective channel gains are $g_{i}={\left|h_{i}^{\mathrm{eff}}\left(\varphi \right)\right|}^{2}$ and $g_{j}={\left|h_{j}^{\mathrm{eff}}\left(\varphi \right)\right|}^{2}$ with $\partial g_{i}/\partial \varphi$ and $\partial g_{j}/\partial \varphi$ given in Appendix A.

Gradients of ${\mathrm{SINR}}_{i,c}$: For the common stream, ${\mathrm{SINR}}_{i,c}=\frac{\alpha P_{i}g_{i}}{\sigma^{2}+\left(1-\alpha \right)P_{i}g_{i}+P_{j}g_{j}}$. Let $F_{c}=\sigma^{2}+\left(1-\alpha \right)P_{i}g_{i}+P_{j}g_{j}$. Then, the gradients are

$$\begin{array}{cc} \frac{\partial {\mathrm{SINR}}_{i,c}}{\partial P_{i}} & =\frac{\alpha g_{i}\left(P_{j}g_{j}+\sigma^{2}\right)}{F_{c}^{2}},\\ \frac{\partial {\mathrm{SINR}}_{i,c}}{\partial P_{j}} & =-\frac{\alpha P_{i}g_{i}g_{j}}{F_{c}^{2}},\\ \frac{\partial {\mathrm{SINR}}_{i,c}}{\partial \alpha } & =\frac{P_{i}g_{i}\left(\sigma^{2}+P_{i}g_{i}+P_{j}g_{j}\right)}{F_{c}^{2}},\\ \frac{\partial {\mathrm{SINR}}_{i,c}}{\partial \varphi } & =\frac{\alpha P_{i}\left(P_{j}g_{j}+\sigma^{2}\right)}{F_{c}^{2}}\frac{\partial g_{i}}{\partial \varphi }-\frac{\alpha P_{i}P_{j}g_{i}}{F_{c}^{2}}\frac{\partial g_{j}}{\partial \varphi }. \end{array}$$

Gradients of ${\mathrm{SINR}}_{j}$: For the strong node’s stream, ${\mathrm{SINR}}_{j}=\frac{P_{j}g_{j}}{\sigma^{2}+\left(1-\alpha \right)P_{i}g_{i}}$. Let $F_{j}=\sigma^{2}+\left(1-\alpha \right)P_{i}g_{i}$. Then, the gradients are

$$\begin{array}{cc} \frac{\partial {\mathrm{SINR}}_{j}}{\partial P_{j}} & =\frac{g_{j}}{F_{j}},\\ \frac{\partial {\mathrm{SINR}}_{j}}{\partial P_{i}} & =-\frac{\left(1-\alpha \right)P_{j}g_{i}g_{j}}{F_{j}^{2}},\\ \frac{\partial {\mathrm{SINR}}_{j}}{\partial \alpha } & =\frac{P_{i}P_{j}g_{i}g_{j}}{F_{j}^{2}},\\ \frac{\partial {\mathrm{SINR}}_{j}}{\partial \varphi } & =-\frac{\left(1-\alpha \right)P_{i}P_{j}g_{j}}{F_{j}^{2}}\frac{\partial g_{i}}{\partial \varphi }+\frac{P_{j}}{F_{j}}\frac{\partial g_{j}}{\partial \varphi }. \end{array}$$

Gradients of ${\mathrm{SINR}}_{i,p}$: For the private stream, ${\mathrm{SINR}}_{i,p}=\frac{\left(1-\alpha \right)P_{i}g_{i}}{\sigma^{2}}$. Then, the gradients are

$$\begin{array}{cc} \frac{\partial {\mathrm{SINR}}_{i,p}}{\partial P_{i}} & =\frac{\left(1-\alpha \right)g_{i}}{\sigma^{2}},\\ \frac{\partial {\mathrm{SINR}}_{i,p}}{\partial P_{j}} & =0,\\ \frac{\partial {\mathrm{SINR}}_{i,p}}{\partial \alpha } & =-\frac{P_{i}g_{i}}{\sigma^{2}},\\ \frac{\partial {\mathrm{SINR}}_{i,p}}{\partial \varphi } & =\frac{\left(1-\alpha \right)P_{i}}{\sigma^{2}}\frac{\partial g_{i}}{\partial \varphi }. \end{array}$$

These analytical gradients enable efficient implementation of the SCA algorithm in the main text.
